# Spoligotyping and Drug Resistance Analysis of *Mycobacterium tuberculosis* Strains from National Survey in China

**DOI:** 10.1371/journal.pone.0032976

**Published:** 2012-03-07

**Authors:** Yu Pang, Yang Zhou, Bing Zhao, Guan Liu, Guanglu Jiang, Hui Xia, Yuanyuan Song, Yuanyuan Shang, Shengfen Wang, Yan-lin Zhao

**Affiliations:** 1 National Center for Tuberculosis Control and Prevention, Chinese Center for Disease Control and Prevention, Beijing, China; 2 Beijing Tuberculosis and Thoracic Tumor Research Institute, Beijing, China; Tulane University, United States of America

## Abstract

**Background:**

Tuberculosis (TB), caused by Mycobacterium tuberculosis complex (MTBC), is one of the major causes of death in the world today. Although China has the second largest global case rate of tuberculosis, a systematic study of TB prevalence in China has not been completed. From 2006 to 2007, the base line surveillance of tuberculosis was carried out by Ministry of Health, and more than 4000 representative strains were selected from 31 provinces in China.

**Methodology/Principal Findings:**

The aim of the present research was to survey the genotypes of representative *Mycobacterium tuberculosis* (*M. tuberculosis*) strains from China using spacer oligonucleotide typing (spoligotyping), and to analyze the relationship between genotype and drug resistance for the first time. A total of 4017 clinical isolates were collected from 2007 to 2008 throughout China. Among those *M. tuberculosis* isolates, 2500 (62.2%) isolates were Beijing genotypes. The percentage of Beijing genotypes in northern China was higher than in southern China (76.5% vs. 53.2%). Additionally, the frequencies of rifampin-resistant, ofloxacin-resistant and multidrug-resistant isolates were significantly higher in Beijing genotype strains than non-Beijing strains. Furthermore, a novel genotype named “China Southern genotype (CS)” was only isolated from Fujian and Guangdong provinces. Hence, it is very practical to uncover the reason for prevalence of the CS type in southern China.

**Conclusions/Significance:**

In conclusion, Beijing family genotypes were still the predominant genotype throughout China, which exhibited a greater correlation with rifampin-resistance, ofloxacin-resistance and MDR phenotypes than other TB spoligotypes, and some regions of China showed several unique characters in the distribution of *M. tuberculosis* genotypes. Our research represents an important contribution for the TB control and research community, which completes broad pictures on drug resistance levels and distribution of *M. tuberculosis* strain types over China.

## Introduction

Tuberculosis (TB), caused by Mycobacterium tuberculosis complex (MTBC), is one of the major causes of death in the world today [1], [2]. It is estimated that one-third of the world's population has been infected by *Mycobacterium tuberculosis*. The situation in developing countries is worse. According to the World Health Organization (WHO), about 95% of the eight million new cases each year occur in middle- and low-income countries [2]. It is well-known that China, where 80% of TB patients live in rural areas [3], has the second largest population of tuberculosis patients [4]. Tuberculosis is not only a disease harming people's health, but also a social-economic issue [3].

Genotyping of *M. tuberculosis* isolates has significantly improved knowledge of the epidemiology of tuberculosis (TB) [5]. With the use of molecular tools, it is possible to detect unsuspected transmission, to identify false-positive cultures, and to distinguish between reinfection and relapse [6]. Spacer oligonucleotide typing (spoligotyping) is a reliable and informative technology for characterizing the genetic structure of tuberculosis populations, especially Beijing family genotype strains [7], [8]. Using PCR technology, spoligotyping analyzes genomic polymorphisms in the short direct repeat (DR) of *M. tuberculosis*, a genetic marker consisting of identical 36-bp DRs interspersed with 35- to 41- bp non-repetitive spacer sequences [8].

Molecular typing of *M.tuberculosis* strains from several Asian countries revealed that a specific family of *M. tb* strains, named the Beijing genotype, were the dominant pattern [9].The Beijing family genotype is also common in the countries of the former Soviet Union [10], but rare in other regions such as Finland and India [11]. In previous reports, the Beijing genotype was found responsible for outbreaks of multidrug-resistant TB (MDR-TB), and some study indicate that Beijing genotype is associated with drug resistance [7], [12]. Although China has the second largest global case rate of tuberculosis (TB), a systematic study of TB prevalence in China, especially Beijing family, has not been completed. Hence, it is meaningful to analyze the situation of Beijing genotype in tuberculosis isolates.

In China, the base line surveillance of tuberculosis was carried out by Ministry of Health recently, and representative strains selected from 31 provinces in China. In this study, we used spoligotyping to classify the 4017 representative strains mentioned above. Our aim was to find the prevalent genotype in China, especially the prevalence of Beijing genotyping. In addition, the relationship between genotype and drug-resistance was also analyzed.

## Results

### Distribution of different genotypes in China

A collection of 4017 representative *M. tuberculosis* isolates was analyzed by spoligotyping in this study. Among these clinical strains, 2500 (62.2%) belonged to the Beijing genotype, while 1515 (37.8%) were non-Beijing family., demonstrating that the Beiijng family is still the predominant genotype in China. Strains classified into non-Beijing family included 182 strains from the MANU2 family (4.5%), 30 from the CAS1-DELH1 family (0.7%), 394 from the T1 family (9.8%), 126 from the T2 family (2.4%), 585 of new found genotype (9.8%) and 200 other (5.0%). According to Chinese Administrative Division, the origin of strains was classified as either northern (1551) or southern (2466) region isolates. The percentage of Beijing genotype in the two regions were 76.5% and 53.2%, respectively, which is a statistically significant difference (χ^2^ = 219.69, P<0.0001). That is, the Beijing genotype shows higher prevalence in the north than south. Prevalence of each of the non-Beijing genotype families was analyzed individually. Prevalence of MANU family genotypes were 3.6% and 5.1%, respectively, a statistically significant value (χ^2^ = 4.95, P = 0.029). The differences of T1 (4.3% vs. 13.3%, χ^2^ = 88.07, P<0.0001), CAS-DELHI (1.9% vs. 0.0%, χ^2^ = 42.98, P<0.0001) and all newly discovered (novel) genotypes (7.1% vs. 19.3%, χ^2^ = 113.34, P<0.0001) were also extremely significant. T2 genotype occupied 2.3% vs. 3.6%, the difference of which was significant. And there was no difference between other genotypes overall (4.3% vs. 5.4%, χ^2^ = 2.32, P = 0.136) (Table 1).

**Table 1:** Distribution of different family strains in Northern and Southern regions.

Region	No.†	No. (%)						
		BEIJING	MANU2	CAS1-DELHI	T1	T2	New found	Others
Total	4017	2500(62.2%)	182(4.5%)	30(0.7%)	394(9.8%)	126(3.1%)	585(14.6%)	200(5.0%)
Northern‡	1551	1187(76.5%)	56(3.6%)	29(1.9%)	66(4.3%)	36(2.3%)	110(7.1%)	67(4.3%)
Southern§	2461	1313(53.2%)***	126(5.1%)*	1(0.0%)***	328(13.3%)***	90(3.6%)*	475(19.3%)***	133(5.4%)

† The number of isolates.

‡ According to Chinese administrative division, the Northern region of China includes provinces as followed: Heilongjiang, Jilin, Liaoning, Inner Mongolia, Hebei, Beijing, Tianjin, Shandong, Henan, Shanxi, Shaanxi, Ningxia, Gansu, Qinghai and Xinjiang.

§ According to Chinese administrative division, the Southern region of China includes provinces as followed: Jiangsu, Anhui, Hunan, Sichuan, Yunnan, Guizhou, Guangdong, Guangxi, Fujian, Jiangxi, Zhejiang, Hainan, Xizang, Shanghai and Chongqing.

* : P<0.05 (significant);

** : P<0.01 (highly significant);

*** : P<0.001 (extremely significant).

### Predominant spoligotypes in China

**Figure 1:**
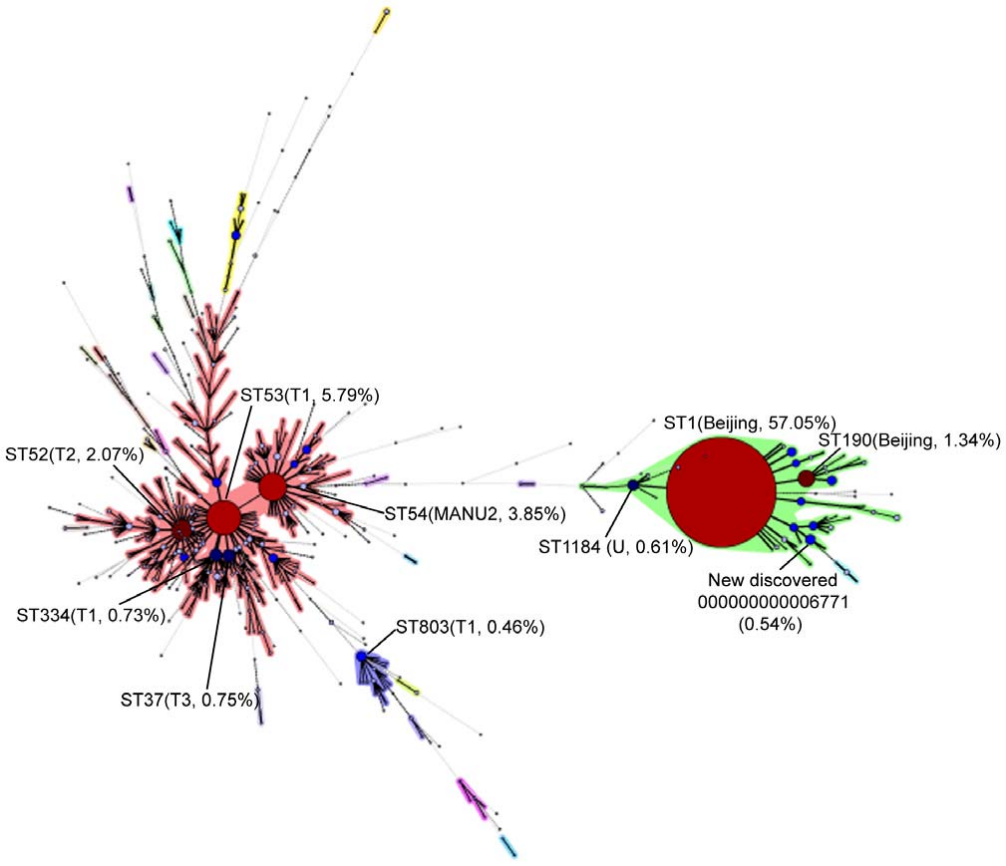
Minimum spanning tree generated with spoligotypes of the respective TB strains from all over the China. Each nodal point represents a particular spoligotype, and the size of nodal point is relative to the number of strains with that spoligotype. The percentage represents the proportion of each spoligotype. The annotations in the figure were the 10 most frequent spoligotypes.

Among the 4017 typed isolates, a total of 568 spoligotypes were identified. Of these, 98 spoligotypes were previously represented as Shared International Types (STs) according to SpolDB4.0, while the other 470 were reported for the first time (Table 2). After clustering with BioNumerics software, 3610 (89.87%) isolates were classified into 161 groups containing 2 or more strains. The mean number of isolates per cluster was 22.42. Additionally, 407 (10.13%) strains did not form clusters (Figure 1, Table 2).

**Table 2:** Numbers and frequencies of isolates clustered by spoligotyping.

Parameter	Value
No. of isolated studied	4017
No. of clusters	161
No. of new found spoligotype	470
Mean no. of isolates per cluster	22.42
No.(%) of clustered isolated	3610 (89.87)
No.(%) of unclustered isolated	407 (10.13)

Of the 161 clusters, about 75% of all isolates were contained in 10 predominant clusters. Nine of these (ST1, ST53, ST54, ST52, ST190, ST37, ST334, ST1184 and ST803) could be found in the SpolDB4.0 database, while the one remaining (spoligotype: 000000000006771) was newly discovered. Among these 10 types, ST1 and ST190 were members of the Beijing family, representing 2343 (57.05%) and 55 isolates (1.34%), respectively. As the second most prevalent type, ST53, the member of T1 family, had 238 (5.79%). Besides, the number of ST54 (MANU2 family), ST52 (T2 family), ST37 (T3 family), ST334 (T1 family), ST1184 (U family) and ST803 (T1 family) were 158 (3.85%), 85 (2.07%), 31 (0.75%), 30 (0.73%), 25 (0.61%) and 19 (0.46%), respectively. The new found spoligotye (000000000006771) included 22 isolates (0.54%). Because this novel type was identified in southern China, it was designated the “CS” (China Southern) type. According to minimum spanning tree, the new CS type was more similar to Beijing genotype than other non-Beijing spoligotypes, and was only found in Fujian and Guangdong provinces, which were both located in southern coastal area of China (Table 3, Figure 1).

**Table 3:** Prevalence of 10 most common Spoligotyping types annotated in SpolDB4.0.

Spoligotype description octonary	SIT*	SpolDB4 ID†	No.‡	Prevalence§ (%)
000000000003771	1	BEIJING	2343	57.05
777777777760771	53	T1	238	5.79
777777777763771	54	MANU2	158	3.85
777777777760731	52	T2	85	2.07
000000000003731	190	BEIJING	55	1.34
777737777760771	37	T3	31	0.75
577777777760771	334	T1	30	0.73
000002000003771	1184	U	25	0.61
000000000006771	NA¶	NA¶	22	0.54
777740007760771	803	T1	19	0.46

* SIT from SpolDB4.0.

† Representing spoligotype families annotated in SpolDB4.0.

‡ Number of strains with the same SIT.

§ Prevalence represents the percentage of isolates with a common SIT among all isolates in this study.

¶ NA represents the spoligotyping type which is not found in SpoIDB4.0.

### Drug susceptibility profiles of Beijing genotype and Non-Beijing genotype

Of 4017 strains with genotyping results in this study, complete drug susceptibility profiles were obtained for 3634 isolates. Of the latter, 2290 (63.0%) were the Beijing genotype, and the other 1344 (37.0%) were non-Beijing genotypes. As shown in Table 4, 744 (20.5%) were INH resistant, 412 (11.3%) were resistant to RIF, 287 (7.9%) were resistant to EMB, 1073 (29.5%) were SM resistant, 85 were resistant to Kan and 145 were OFLX resistant. Of these strains, 62 and 27 isolates were designated MDR (9.9%) and XDR (0.7%), respectively. The DST results comparison between Beijing family and non-Beijing family showed that the percentages of RIF resistant (21.7% vs. 18.4%), OFLX resistant (4.9% vs. 2.4%) and MDR (11.3% vs. 7.4%) in Beijing genotype were extremely significantly higher than those of non-Beijing genotype, the chi-square value of which were (χ^2^ = 22.10, P<0.0001), (χ^2^ = 14.42, P<0.0001) and 7.4% (χ^2^ = 14.83, P<0.0001), severalty. Similarly, Beijing family provided higher proportion in INH resistant (21.7% vs. 18.4%, χ^2^ = 5.75, P = 0.017) and EMB (8.8% vs. 6.4%, χ^2^ = 6.59, P = 0.011) resistant than non-Beijing family. In contrast, the ratio of SM resistant in non-Beijing genotype (32.4%) was higher than that of Beijing (27.9%), the difference of which was highly significant (χ^2^ = 8.26, P = 0.004). The percentage of Kan resistant was strikingly similar for both genotypes. Because the number of XDR isolates identified was small, there was no statistical difference in XDR frequencies between Beijing and non-Beijing genotypes (Table 4).

**Table 4:** Prevalence of antituberculosis drug resistance among 3634 isolates in China TB survilliance by genotype.

Genotype	% Resistance to†:							
	Isonazid	Rifampin	Ethambutol	Streptomycin	Kanamycin	Ofloxacin	MDR‡	XDR§
Beijing (n = 2290)	21.7	13.2	8.8	27.9	2.2	4.9	11.3	1.0
Non-Beijing (n = 1344)	18.4*	8.1***	6.4*	32.4**	2.5	2.4***	7.4***	0.4
Odds ratio	1.23	1.73	1.41	0.81	0.88	2.13	1.60	2.60
95% CI	1.04–1.46	1.37–2.18	1.08–1.83	0.70–0.93	0.57–1.36	1.43–3.17	1.26–2.04	0.98–6.88

† Isolates resistant to isonazid (*n* = 744; 20.5%), rifampin (*n* = 412; 11.3%), ethambutol (*n* = 287; 7.9%), streptomycin (*n* = 1073; 29.5%), kanamycin (*n* = 85; 2.3%), ofloxacin (*n* = 145; 4.0%), multiple drugs (MDR) (*n* = 62; 9.9%) or extensively drugs (XDR) (*n* = 27; 0.7%).

‡ MDR, multidrug resistant, represents isolates resistant to at least isonazid and rifampin.

§ XDR, extensively drug resistant, represents isolates resistant to at least rifampicin and isoniazid, as well as any member of the quinolone family and at least one of the following second-line anti-TB injectable drugs.

* : P<0.05 (significant);

** : P<0.01 (highly significant);

*** : P<0.001 (extremely significant).

### Drug susceptibility profiles of predominant spoligotypes in China

The five most predominant spoligotypes as well as the newly described CS spoligotype were selected to determine drug susceptibility profiles. The ST1 type, a member of Beijing family, was designated as the control, for comparison with other types. As shown in Table 5, relative to the ST1 profile, ST53 and ST54 presented bigger difference than other types. As the second most frequent strain, ST53 displayed lower INH (14.0% vs. 21.7%, χ^2^ = 7.55, P = 0.006), RIF (6.0% vs. 13.2%, χ^2^ = 8.76, P = 0.002) and OFLX (0% vs. 4.6%, χ^2^ = 8.22, P = 0.001) resistance. The frequency of MDR isolates with the ST53 genotype was significantly lower than that of ST1 (6.0% vs. 11.2%, χ^2^ = 5.24, P = 0.020), which contributed greatly to low drug resistant percentage of non-Beijing family. On the contrary, the frequency of drug resistance was higher in ST54 than ST1, and the proportions of EMB (15.9% vs. 8.6%, χ^2^ = 7.58, P = 0.010), INH (31.8% vs. 21.7%, χ^2^ = 6.88, P = 0.011) and SM (42.9% vs. 27.8%, χ^2^ = 13.26, P = 0.001) resistant were significantly higher. Besides, there were no RIF resistant and MDR isolate in ST52 type, therefore, the ratio of them is lower than those of ST1 significantly (Table 5).

**Table 5:** Prevalence of antituberculosis drug resistance among the most popular genotypes in China TB survilliance.

SIT†	SpolDB4 ID‡	No.§	% Resistance to :						
			Isonazid	Rifampin	Ethambutol	Streptomycin	Kanamycin	Ofloxacin	MDR¶
1	BEIJING	2153	21.7	13.2	8.6	27.8	2.0	4.6	11.2
53	T1	212	14.0**	6.0**	5.0*	33.0	2.0	0**	6.0*
54	MANU2	126	31.8**	15.9	15.9**	42.9***	4.0	4.0	15.1
52	T2	76	21.1	0**	2.6	31.6	0	2.6	0**
190	BEIJING	51	11.8	5.9	5.9	31.4	3.9	0	3.9
New	New	20	25.0	15.0	10.0	20.0	0	10.0	15.0

† SIT from SpolDB4.0.

‡ Representing spoligotype families annotated in SpolDB4.0.

§ Number of isolates with both the same SIT and results of drug sensitive test.

¶ MDR, multidrug resistant, represents isolates resistant to at least isonazid and rifampin.

* : P<0.05 (significant);

** : P<0.01 (highly significant);

*** : P<0.001 (extremely significant).

## Discussion

China has one of the highest tuberculosis burdens in the world. In previous reports, genotyping was performed on some clinical TB isolates collected from several areas of China, which provided us the knowledge about the distribution of dominant TB genotypes. However, due to incomplete representation, these strains could not reveal prevalence and epidemic trend of tuberculosis in China. In this study, 4017 strains were enrolled from the National Drug Resistance Base-Line Surveillance of Tuberculosis. Of these, 62.2% were representatives of the Beijing genotype. Early research based on clinical isolates of different region showed that the percentage of Beijing genotype varied from 67.1% to 92% [7], [11], [13]–[17], which was higher than our research. The explanation of this obvious difference may be that the strains used in previous study were obtained from clinical hospital instead of epidemiological survey. In comparison with surrounding countries, prevalence of the Beijing genotype of this study is less than Hong Kong (70%) [18], but more than Taiwan (44.4%) [19], Vietnam (54%) [20], Thailand (44%) [21] and Russia (44.5%) [22]. Hence, this study demonstrates that the Beijing genotype is a dominant TB genotype of China.

The spoligotying profiles of different geographical areas including north and south China showed different characteristics. The main diversity was that the proportion of Beijing genotype in the north area was higher than that in the south area. However, the T family genotype, one of the prevalent genotypes in Africa, Central and South America and Europe [23], was significantly higher in southern China than in the north. In addition, more novel spoligotypes were identified in southern China. Overall, the genetic variability of the DR locus was more diversified in the south of China, and several reasons may be responsible for it. First of all, the economic state and traffic condition of southern is better than that of northern, causing more frequent movement of the population between either different provinces or different countries in south area. Additional, the dissimilar customs adapted to the each climate may the other possible reason for this diversity. People lived in the north China will share longer indoor time because of cold winter months, which may be related with the prevalence of Beijing genotype. However, the proof of this hypothesis is difficult since the more population-based epidemical results are necessary. Interestingly, CAS1-DEHL1 type was almost exclusively detected in northern China (29/30). Of the 29 isolates, 26 were collected from Xinjiang Uygur Autonomous Region. In a previous report, the Middle East, and Central and Southern Asia were “high incidence” areas of CAS1-DEHL1 family [23]–[25]. Obviously, the Xinjiang region is adjacent to several Central Asian countries and they share similar culture traditions. Hence, frequent movement of ethnic groups may be the major factor to explain the prevalence of the CAS1-DEHL1 genotype in Xinjiang [26].

According to subtype analysis with Shared International Types (SITs), a novel CS genotype containing 22 strains was all indentified among the isolates from Fujian and Guangdong provinces, which were all located in the south area of China. Hence, it is very practical to uncover the reason for prevalence of the CS type in southern China. In general, as the high prevalence of this genotype may be related to enhanced virulence, transmissibility, and/or specific adaptation to a host population, isolates with the CS genotype need to be studied carefully.

According to literature reports, the Beijing genotype has significant associations with drug-resistance and might be responsible for the spread and emergence of MDR-TB. Studies in several countries including Vietnam, Germany, Cuba and America, have confirmed these results. Because of the high incidence of the Beijing genotype in many regions, some researchers have proposed that the Beijing genotype may be correlated with high virulence and easy transmission. In order to define any association of drug resistance with the Beijing genotype, we determined the frequency of the Beijing genotype among bacterial isolates exhibiting different categories of drug resistance. In this study, comparison between Beijing genotype and non-Beijing genotype, the resistance to RIF and OFLX was both significantly higher in Beijing, In agreement with previous reports, the proportion of MDR-TB was also higher in Beijing genotypes compared to non-Beijing families. In 2006, Nikolayevskyy et al. revealed that the Beijing genotype was closely related to RIF and INH resistance as well as to MDR in the Ukraine [27]. Research in Central Asia showed similar results, including higher RIF, INH, SM and MDR resistance in Beijing family [12]. Referred to OFLX, Duong et al. reported that gyrA mutations were more common in Beijing type, which resulted in higher levels of OFLX resistance [28]. In contrast to previous studies [20], we found that the non-Beijing genotype presented higher SM resistance than Beijing. Our results are in agreement with studies performed by Niemann *et al.* [29] and Tanveer et al. [30], respectively, which showed that some non-Beijing genotypes represented higher percentage of SM-resistance. It is obvious that non-Beijing genotype comprised of a large number of sub-genotypes. Hence, the conflicting results observed in different studies may be due to the different distribution of non-Beijing genotypes. While the association between Beijing genotype and levels of drug resistance appear to arise from high mutation rates in drug resistance-associated genes, it was not determined whether virulence and infectivity of the Beijing family was more critical than other genotypes. That is because the discriminatory power of spoligotyping is not ideal, and the strains selected as the Beijing type may not represent for Beijing family well.

Some published studies have revealed several families of non-Beijing genotypes, such as Haarlem family [31] and LAM family [32], have also shown affinity to drug resistance. Therefore, it is meaningful to analyze the relationship between spoligotype and drug-resistant phenotypes in China. After analysis towards the fifth most popular and new found (CS) types, we can find that ST1, the main member of Beijing genotype in China, was responsible for the strong association between the Beijing genotype and resistance to several drugs as well as with MDR-TB. In this investigation we also found a high proportion of drug resistance in ST54 genotypes from the MANU2 family. The level of INH, EMB and SM resistance for ST54 was even greater than ST1. The MANU2 family has a low prevalence worldwide, so there are few reports on an association between phenotype and the MANU2 genotype. In a study from South Africa, 2 isolates were enrolled, one of which was resistant to RIF, and the other of which was INH resistant. As this is the first report of a strong association between drug resistance and the MANU2 family genotype, further confirmatory research is needed. In addition, the drug resistance of CS type displayed similar patterns with ST1, which also indicated that CS type was a member of Beijing family, and might derive from ST1.

This study shows the prevalence of different TB genotypes in China for the first time, which completes broad pictures on drug resistance levels and distribution of *M. tuberculosis* strain types over China, and Beijing genotype (62.2%) is still the predominant strains in China, while the percentage of the type is lower than previous report. The distribution of Beijing family represents that the proportion of north is significant higher than that of south. Due to the characters of population mobility in China, the composition of other spoligotypes also shows distinct features. Because of the limitation of spoligotyping, future studies are needed to perform genotyping with more powerful technologies to consummate the genotype data, such as MIRU-VNTR and RFLP. Moreover, Beijing family is found to be significantly associated with RIF and OFLX resistance, as well as with MDR-TB, and it is essential to carry out a systematic study on relationship between genotype and phenotype of Beijing genotype.

## Materials and Methods

### Study population and bacterial strains

**Figure 2:**
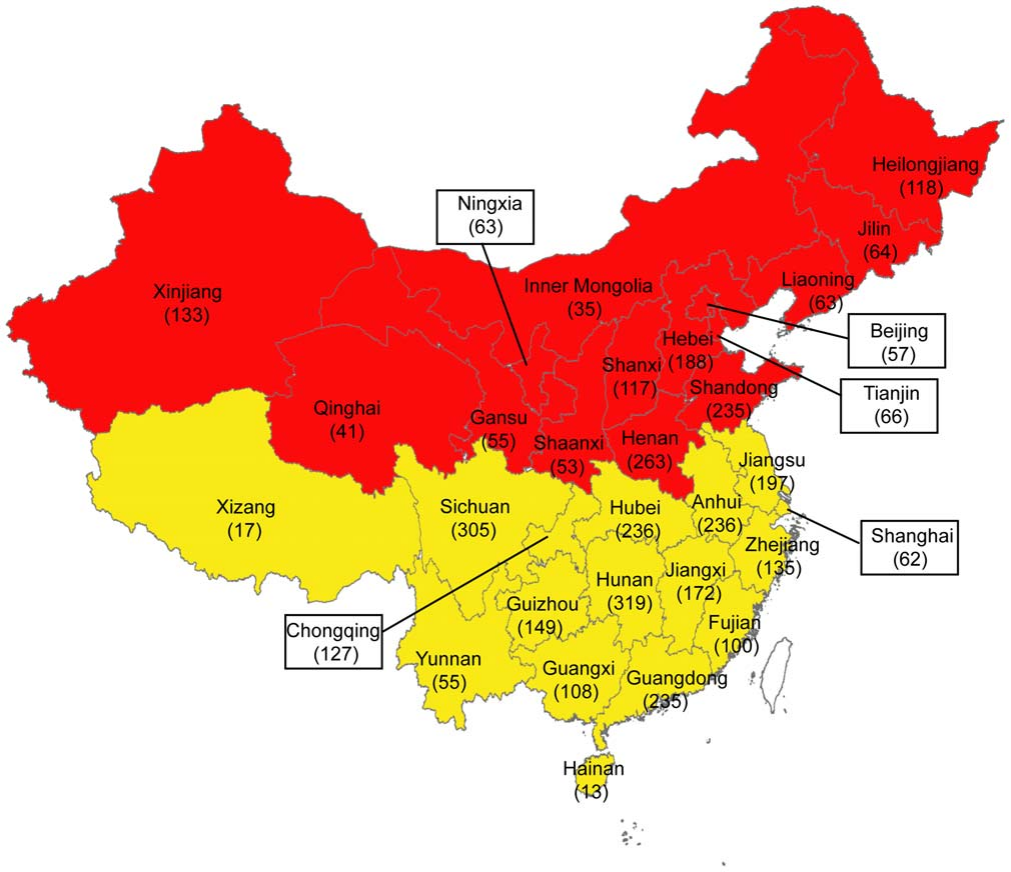
Distribution map of M. tuberculosis isolates included in this study. The provinces colored with red represent the northern region of China including Heilongjiang, Jilin, Liaoning, Inner Mongolia, Hebei, Beijing, Tianjin, Shandong, Henan, Shanxi, Shaanxi, Ningxia, Gansu, Qinghai and Xinjiang. And the provinces colored with yellow represent the southern region of China including Jiangsu, Anhui, Hunan, Sichuan, Yunnan, Guizhou, Guangdong, Guangxi, Fujian, Jiangxi, Zhejiang, Hainan, Xizang, Shanghai and Chongqing.

A total of 4017 clinical strains of tuberculosis, isolated from the base-line surveillance of tuberculosis in 2006, were collected from 31 provinces in China excluding Taiwan, Hong Kong and Macao. The distribution of isolates was shown in Figure 2. The number of patients enrolled in surveillance was proportionally based on patient numbers in different provinces. The protocols applied in this study were approved by the Ethics Committee of the Chinese Center for Disease Control and Prevention. Patients were able to access the study if they signed an Informed Consent form. All strains, stored in Trypticase soy broth with glycerol ay −70°C freezer, were recovered on Lowenstein-Jensen medium for 4 weeks at 37°C.

### Drug susceptibility testing and Mycobacterium species identification

Mycobacterium identification and testing of the drug susceptibility of these strains to four first-line anti-TB drugs (isonazid, rifampin, ethambutol and streptomycin) and two second-line anti-TB drugs (kanamycin and ofloxacin) were performed as recommended by WHO/IUATLD [33]. The concentrations of drugs in media were as following: isonazid 0.2 µg/mL, rifampin 40 µg/mL, ethambutol 2 µg/mL, streptomycin 4 µg/mL, kanamycin 30 µg/mL and ofloxacin 2 µg/mL. Strains resistance to isoniazid and rifampin were defined as MDR-TB. The strains was declared resistant to the specific drug when the growth rate was >1% compared to the control. In addition, XDR-TB was defined as isolates resistant to rifampicin and isoniazid, as well as any member of the quinolone family and at least one of the following second-line anti-TB injectable drugs. Media supplied separately with paranitrobenzoic acid (500 mg/mL) and thiophen-2-carboxylic acid hydrazide (5 mg/mL) was used to perform Mycobacterium species identification. All the drugs were purchased from Sigma-Aldrich (St. Louis, MO).

### Molecular typing methods

Genomic DNA was extracted from freshly cultured bacteria. Cells were resuspended in 500 µL TE buffer(pH 8. 0), then heated in 95°C water bath for 1 hour. After centrifugation of cellular debris, DNA in the supernatent served as PCR template. Spoligotyping was used to identify the genotype of TB strain in the DR locus as described previously [10]. A commercially available kit (Isogen Bioscience BV, Maarssen, The Netherlands) was used as described by the manufacturer. All chromosomal DNA was amplified with primers DRa (5′-CCGAGAGGGGACGGAAAC-3′) and DRb (5′-GGTTTTGGGTCTGACGAC-3′). Then the amplified products were hybridized with the membrane. After washing with 2×SSPE solution (360 mM NaCl, 20 mM NaH_2_PO_4_, 2 mM EDTA [pH 7.2]) supplemented with 0.5% SDS, the membrane was hybridized with streptavidin-peroxidase conjugate. The final image was detected with a chemiluminescence system, including the ECL detection liquid (Amersham, Buckinghamshire, United Kingdom) and ECL-Hyperfilm (Kodak, Rochester, NY). Beijing genotype strains were defined with the pattern that hybridized to all of the last nine spacer oligonucleotides (spacers 35 to 43), and Beijing-like genotype strains were ones that hybridized to only some of the last nine spacers.

### Data analysis

The genotyping were expressed in binary and octal formats in Microsoft Excel spreadsheets. Drug resistance test results were arranged in binary formats in Excel. All spoligotype data was submitted to the international databases MIRU-VNTRplus(http://www.miru-vntrplus.org) [23] and SpolDB4.0 http://www.pasteur-guadeloupe.fr:8081/SITVITDemo) [13]. In addition, the resulting data was analyzed using BioNumerics (Version5. 0, Applied Maths, Sint-Martens-Latem, Belgium) software. Cluster analysis was performed and a dendrogram was generated in Bionumerics using the Dice similarity coefficient and UPGMA coefficient. In addition, statistical analyses between genotype and phenotype were performed in SPSS 11.5 (SPSS Inc.). Chi-square and Crosstab analysis were run to identify if the differences between two statistical datas were significant, and P value less than 0.05, 0.01 and 0.001 were defined as “significant”, “highly significant” and “extremely significant” diversity, respectively. The extent of association was shown as an odds ratio (OR) and 95% confidence interval (95% C.I.).
